# COVID-19 and the re-opening of schools: a policy maker’s dilemma

**DOI:** 10.1186/s13052-020-00844-1

**Published:** 2020-06-09

**Authors:** Maria Pia Fantini, Chiara Reno, Giovanni Battista Biserni, Elena Savoia, Marcello Lanari

**Affiliations:** 1 Department of Biomedical and Neuromotor Sciences, Alma Mater Studiorum - University of Bologna, 40126 Bologna, Italy; 2 Department of Pediatric Emergency Unit, S. Orsola Hospital, Department of Medical and Surgical Sciences, Alma Mater Studiorum - University of Bologna, 40138 Bologna, Italy; 3 Emergency Preparedness Research Evaluation & Practice Program Harvard T.H. Chan School of Public Health, Boston, MA 02115 USA

**Keywords:** COVID-19, SARS-CoV-2, Children, Health policy, School closure, Lockdown

## Abstract

The epidemic of coronavirus disease 2019 (COVID-19) broke out in Wuhan, China, in December 2019 and rapidly spread across the world. In order to counter this epidemic, several countries put in place different restrictive measures, such as the school’s closure and a total lockdown. However, as the knowledge on the disease progresses, clinical evidence showed that children mainly have asymptomatic or mild disease and it has been suggested that they are also less likely to spread the virus. Moreover, the lockdown and the school closure could have negative consequences on children, affecting their social life, their education and their mental health. As many countries have already entered or are planning a phase of gradual lifting of the containment measures of social distancing, it seems plausible that the re-opening of nursery schools and primary schools could be considered a policy to be implemented at an early stage of recovery efforts, putting in place measures to do it safely, such as the maintenance of social distance, the reorganisation of classes into smaller groups, the provision of adequate sanitization of spaces, furniture and toys, the prompt identification of cases in the school environment and their tracing. Therefore, policy makers have the task of balancing pros and cons of the school re-opening strategy, taking into account psychological, educational and social consequences for children and their families. Another issue to be considered is represented by socio-economic disparities and inequalities which could be amplified by school’s closure.

## Main text

Italy was the first European country to implement a national lockdown to contain the spread of severe acute respiratory syndrome coronavirus 2 (SARS-CoV-2) and mitigate the impact of an inevitable surge of COVID-19 cases. After over 8 weeks of social distancing measures, the country is now shifting its strategy from mitigation to recovery, and other countries are watching how Italy will re-open and contain new clusters, with the hope of learning from its experience.

In children, the diagnosis of COVID-19 is complex due to lack of specificity of its symptoms (fever, fatigue, and dry cough), causing difficulties in the differential diagnosis with pediatric infectious diseases occurring in winter and spring seasons. Moreover, children are often unable to describe minor symptoms related to this new disease, for instance myalgia, headache, anosmia and ageusia, and cases can be easily missed. In fact, as observed in a series of 731 pediatric COVID-19 cases the cumulative incidence of patients with asymptomatic, mild or moderate disease was 97%, suggesting a milder presentation in children [1]. This finding is consistent with the results of a systematic review, which found that children at any age were mostly reported to have mild symptoms or were asymptomatic and that pediatric patients with COVID-19 had generally a good prognosis and recovered within 1 or 2 weeks after disease onset [2].

As the pandemic progresses, more data are becoming available on how different age segments of the population are susceptible to the infection. Considering recent data [3], we calculated the cumulative incidence in the three Italian regions most affected by the epidemic, Lombardy, Emilia-Romagna and Veneto, that are 0.29, 0.34 and 0.34 per 1000 children 0–9 years old, respectively, lower compared to the rest of the population. These data are also consistent with reports from the Republic of Korea [4] where only 1% of the first 7755 laboratory-confirmed cases occurred in the 0–9 age group. Additionally, a testing of at risk individuals in Iceland through oro- and nasopharyngeal swabs showed that children under 10 years of age were less likely to test positive (6.7%) compared to other age groups, and that in the general population screening no child under 10 years of age resulted positive [5]. In the municipality of Vo’ (Veneto region, Italy), where the first Italian related COVID-19 death was registered, the entire population was tested twice for the presence of SARS-CoV-2 with nasopharyngeal swabs and no infections were detected in the 234 children aged 0–10, despite at least 13 of them were living with infected family members [6]. A recent investigation suggests that the spread of COVID-19 within New South Wales (Australia) schools has been very limited. In particular, on a total of initial six cases in five primary schools (one student and five staff), only one of 168 close contacts was identified as a secondary case. Moreover, the Australian report shows a small probability of infection among children and no evidence of children infecting teachers [7]. Otherwise, severe cases of SARS-CoV-2 infection in children under 2 years old have been reported in literature [1, 2] and recently, several cases of a multisystem inflammatory syndrome in children with a possible temporal association with SARS-CoV-2 infection have been reported even if further investigations are needed to confirm the association with SARS-CoV-2 [8].

In light of such data, a thoughtful consideration of the implications of school closure policies on children’s health is necessary. Children aged from 2 to 10 years old have an active social life at school which helps learning from peers and positively impacts the development of personality traits and sense of identity. Not only, disruptions of close peer relationships have been associated with depression, guilt, and anger in children. In addition, children experiencing isolation and quarantine have shown an increased risk of developing post-traumatic stress disorder, anxiety, grief, and adjustment disorder [9]. Parents are often the only care providers for children, which limits their work productivity, even when they are fortunate to have a job that allows them to work from home. In some cases, forced cohabitation in a home environment, with parents suffering from economic and mental health issues exposes children to the risk of uncovering violent behaviors. Regarding the educational aspects, during the lockdown, e-learning is not always a feasible alternative to face-to-face instruction for these aged children, particularly when acquiring hand-eye coordination for writing. E-learning could also amplify inequalities (digital divide). Therefore, the potential benefits of dismissing students aged 2 to 10 years old from schools to contain the spread of infection may be outweighed by the negative consequences of keeping them home.

The questions being asked are what could the conditions necessary for a safe opening of schools for children aged from 2 to 10 years be and secondly can school re-opening be considered as one of the policies to be implemented at an early stage in recovery efforts? [10]. As shown before, children aged from 2 to 10 years have a low rate of severe infection, a probably marginal role in spreading the disease, but at the same time they have a big toll to pay for school closure. It seems plausible that the re-opening of nursery and primary schools can be considered a policy to be implemented at an early stage of recovery efforts, but it is important to be able to guarantee safe conditions and an appropriate surveillance system. Safe measures for the re-opening of the schools may include the creation of fixed small groups of children, in order to balance the need to go to school and the need to maintain social distance, taking into account the available spaces and potentially considering the implementation of differentiated shifts to attend schools. Avoidance the sharing of materials, reallocation of common rooms and areas, together with ensuring frequent access to hand washing could also represent successful strategies that can be modulated according to the organisational capacity of the single institution. Measures such as ventilation of rooms and sanitization of environments are fundamental. Moreover, children could greatly benefit from time spent outdoors. In order to check the feasibility of this approach, in the first phase, partial class re-opening, coupled with e-learning could be provided. Teaching and school staff should be additionally trained to identify early signs of mental health issues related to quarantine and isolation. Concerning surveillance system, this should consist in proper information/education of teachers and parents, prompt identification of cases in the school environment, testing capacities, case tracing, isolation, and quarantine.

In Denmark, where the public health system showed the capacity to promptly identify and trace COVID-19 cases, the Government took the decision to send back to school children up to 11 years old on April 15, 2020. School activities have been properly designed in order to limit as much as possible any spread of the virus. After a month, the adopted measures (the creation of small groups of children for lessons and for playtime, frequent hand washing, student’s desks spaced 6 feet apart, and, whenever possible, classes held outside) seem to be effective. In Japan, schools have reopened following the proposal of the Government to give priority for some grades, including first- and sixth- grader at elementary schools. In particular, the decision on when and whether or not to reopen schools has been left to local municipalities based on the number of COVID-19 cases in the area. Guidelines for schools re-opening have been released by the Ministry of Health. The included measures range from checking temperature daily, to maintaining physical distance and wearing face masks. We do not have by now information about safety and effectiveness of school re-opening in Japan.

To sum up, the strategies of schools’ re-opening, if implemented, taking into account the balance of pros and cons for children of the mentioned age, should be led by a flexible approach in order to adapt to the local context in terms of epidemiological data and system capabilities.

## Data Availability

Not applicable.

## Abbreviations

COVID-19: Coronavirus disease 2019
SARS-CoV-2: Severe acute respiratory syndrome coronavirus 2
